# Single-Center Real World Study of Everolimus and Exemestane in HR+/HER2− Metastatic Breast Cancer Following CDK4/6 Inhibitor Therapy

**DOI:** 10.3390/curroncol32090503

**Published:** 2025-09-10

**Authors:** Yunus Emre Altıntaş, Oğuzcan Kınıkoğlu, Deniz Işık, Aziz Batu, Ayberk Bayramgil, Büşra Niğdelioğlu, Uğur Özkerim, Sıla Öksüz, Heves Sürmeli, Nedim Turan, Hatice Odabaş

**Affiliations:** 1 Department of Medical Oncology, Kartal Dr. Lütfi Kırdar City Hospital, Istanbul 34865, Türkiye; ogokinikoglu@yahoo.com (O.K.); dnz.1984@yahoo.com (D.I.); ugur.ozkerim@hotmail.com (U.Ö.); sila.oksuz@gmail.com (S.Ö.); hevessurmeli@hotmail.com (H.S.); turan.nedim@hotmail.com (N.T.); odabashatice@yahoo.com (H.O.); 2 Department of Medical Oncology, Ümraniye Training and Research Hospital, Istanbul 34760, Türkiye; azizbatu84@gmail.com (A.B.); ayberkbayramgil@gmail.com (A.B.); busranigdelioglu@gmail.com (B.N.)

**Keywords:** everolimus, exemestane, CDK4/6 inhibitor resistance, mTOR inhibition, advanced breast cancer

## Abstract

Background: Hormone receptor-positive (HR+), HER2− negative metastatic breast cancer (MBC) is the most common subtype of advanced breast cancer. Resistance to endocrine therapy often develops, particularly after CDK4/6 inhibitors. Everolimus, an mTOR inhibitor, may restore hormone sensitivity, but real-world data after CDK4/6 and chemotherapy are limited. Methods: This retrospective, single-center study included 70 patients with HR+/HER2− MBC who progressed on CDK4/6 inhibitors and at least one line of chemotherapy. All received daily oral everolimus (10 mg) plus exemestane (25 mg). Tumor response was assessed via RECIST v1.1, and survival outcomes were estimated using the Kaplan–Meier method. Results: Median progression-free survival was 6.6 months and overall survival was 22.6 months. The disease control rate was 88.6%, with 57.1% showing partial response. Fatigue (20%), skin toxicity (8.6%), and stomatitis (5.7%) were the most common adverse events. No grade 3–4 toxicities or discontinuations occurred. No clinical or pathological variables significantly influenced survival. Conclusions: Everolimus plus exemestane provided meaningful clinical benefit and manageable toxicity in heavily pretreated HR+/HER2− MBC patients. This regimen remains a valid later-line option, particularly in settings with limited access to newer targeted therapies or genomic testing.

## Simple Summary

Hormone receptor-positive breast cancer is the most common type of advanced breast cancer in women. While hormone treatments are usually effective, many patients eventually stop responding. In this real-world study, we looked at the use of two oral drugs, everolimus and exemestane, in women whose cancer had already progressed after modern treatments like CDK4/6 inhibitors and chemotherapy. We found that this drug combination helped control the disease in nearly 9 out of 10 patients and was generally well tolerated, with few serious side effects. Our results suggest that everolimus and exemestane can still offer meaningful benefits even in later stages of treatment, especially when newer targeted drugs are not available. These findings may help guide treatment decisions and support the continued use of this combination in everyday clinical practice.

## 1. Introduction

Hormone receptor-positive (HR+), human epidermal growth factor receptor 2-negative (HER2−) metastatic breast cancer (MBC) represents the most prevalent subtype of advanced breast cancer in postmenopausal women. Although endocrine therapy remains the cornerstone of treatment in this group, resistance either de novo or acquired poses a major therapeutic challenge and limits the long-term efficacy of hormonal agents such as selective estrogen receptor modulators (SERMs), selective estrogen receptor degraders (SERDs), and aromatase inhibitors (AIs) [1,2].

AIs, including anastrozole, letrozole, and exemestane, are particularly favored in postmenopausal patients due to their superior efficacy compared to tamoxifen [3,4]. Exemestane, a steroidal irreversible aromatase inactivator, has demonstrated benefit both as a first-line agent and as a sequential option following nonsteroidal AI therapy [4]. However, progression under AI treatment is common, emphasizing the need for mechanisms to overcome endocrine resistance [1,5].

Multiple studies have elucidated that one key pathway implicated in this resistance is the phosphatidylinositol 3-kinase/protein kinase B/mammalian target of rapamycin (PI3K/AKT/mTOR) signaling cascade [6,7]. This pathway regulates cellular proliferation, metabolism, angiogenesis, and survival, and is often aberrantly activated in HR+ breast tumors. In particular, mTOR (mammalian target of rapamycin) plays a central role in integrating nutrient and growth factor signals, and its inhibition has emerged as a promising strategy to restore hormone sensitivity [6,8].

Everolimus, an oral mTOR inhibitor, was evaluated in combination with exemestane in the BOLERO-2 study—a pivotal randomized phase III trial—which demonstrated significantly improved progression-free survival (PFS) in patients who had previously progressed on nonsteroidal AIs [7]. This combination received regulatory approval and has since become an established treatment option in endocrine-pretreated HR+/HER2− MBC [5,7]. Further data from preclinical and translational studies also suggest that mTOR inhibition might suppress tumor growth by modulating hypoxia signaling and autophagy through hypoxia-inducible factor 1-alpha (HIF-1α), underscoring a broader biological rationale for its use [8].

Despite the evidence from clinical trials, real-world data remain limited, especially in regions with unique healthcare reimbursement frameworks. In Turkey, for instance, the national reimbursement authority mandates that everolimus plus exemestane therapy can only be reimbursed in patients with HR+/HER2− MBC who have failed prior treatment with nonsteroidal AIs and received at least one line of chemotherapy in the metastatic setting [9]. This constraint may influence treatment sequencing and patient selection and necessitates local data to guide clinical practice.

Given this context, the present study aims to evaluate the efficacy and safety of everolimus combined with exemestane in a real-world cohort of postmenopausal patients with HR+/HER2− MBC treated in accordance with national reimbursement criteria. By providing insights from retrospective clinical experience, this analysis contributes to the evolving understanding of endocrine resistance and the therapeutic role of mTOR inhibition in advanced breast cancer.

## 2. Materials and Methods

This retrospective, single-center study included premenopausal and postmenopausal women diagnosed with HR+, HER2− de novo MBC who were treated at Kartal Dr. Lütfi Kırdar City Hospital (Istanbul, Turkey) between January 2020 and December 2024. Eligible patients had received first-line therapy with a cyclin-dependent kinase 4/6 (CDK4/6) inhibitor in combination with endocrine therapy. In accordance with national reimbursement regulations, all patients subsequently received at least one line of chemotherapy in the metastatic setting prior to initiation of everolimus-based treatment. Everolimus plus exemestane was initiated following progression on chemotherapy.

Inclusion criteria were as follows: (1) histologically confirmed diagnosis of HR+/HER2− de novo MBC; (2) premenopausal or postmenopausal status at diagnosis; (3) receipt of first-line therapy with a CDK4/6 inhibitor plus endocrine therapy; (4) administration of at least one line of chemotherapy before everolimus-based treatment; (5) initiation of everolimus at 10 mg/day and exemestane at 25 mg/day; and (6) availability of complete clinical, treatment, and follow-up data. De novo MBC was defined as the presence of metastatic disease at the time of the initial breast cancer diagnosis. Patients who developed metastatic relapses following prior curative surgery or adjuvant systemic therapy were excluded. Exclusion criteria included HER2-positive or triple-negative breast cancer, prior exposure to mTOR inhibitors, incomplete or missing medical records, and a history of another active malignancy within the last five years. A total of 70 patients who fulfilled these criteria were included in the final analysis.

All clinical data were retrospectively retrieved from the institutional electronic medical records system. Estrogen receptor (ER) status was determined by immunohistochemistry (IHC), and tumors with ≥1% nuclear staining were classified as ER-positive, in line with ASCO/CAP recommendations. HER2 status was determined by IHC. Patients with IHC 0 were classified as HER2-negative, whereas patients with IHC 1+ or IHC 2+ and ISH-negative were categorized as HER2-low. In this study, both HER2-negative and HER2-low patients were included in the analysis, consistent with the current clinical categorization of HER2-low within the HER2-negative spectrum. Genetic data such as BRCA1/2 mutational status were not routinely available in the institutional records and therefore were not included in the present analysis. Everolimus was administered orally at a daily dose of 10 mg, and exemestane at 25 mg once daily, following the dosing strategy adopted in the BOLERO-2 trial. Treatment was continued until radiologically confirmed disease progression, intolerable toxicity, or voluntary discontinuation. The index date was defined as the initiation of everolimus plus exemestane therapy. None of the patients classified as HER2-low received trastuzumab deruxtecan, as this agent was not reimbursed or available in Türkiye during the study period. Radiological assessments were performed at baseline and subsequently at regular intervals of approximately every 8–12 weeks, or earlier if clinically indicated, to evaluate treatment response according to Response Evaluation Criteria in Solid Tumors (RECIST) version 1.1 [10]. This schedule ensured repeat assessment and minimized censoring for progression-free survival analyses. Patients were followed until disease progression, death, or last known follow-up.

All statistical analyses were performed using SPSS Statistics version 26.0 (IBM Corporation, Armonk, NY, USA). Descriptive statistics were used to summarize baseline demographic and clinical characteristics. Continuous variables were expressed as means with standard deviations or medians with interquartile ranges, while categorical variables were summarized as frequencies and percentages. PFS was defined as the time from the initiation of everolimus plus exemestane therapy to radiologically confirmed disease progression or death from any cause. Overall survival (OS) was defined as the time from the same index date to death from any cause. Both survival endpoints were estimated using the Kaplan–Meier method. Median survival times and corresponding 95% confidence intervals were reported. As this was a single-arm study, no formal comparisons between treatment groups were performed. The proportional hazards assumption for Cox regression was tested using Schoenfeld residuals and was not violated. Categorical variables (HER2 status, menopausal status, and ECOG performance status) were coded as binary factors.

The study protocol was reviewed and approved by the Institutional Review Board of Kartal Dr. Lütfi Kırdar City Hospital (Approval No: 2025/010.99/1611; Date: 27 May 2025). All procedures involving human participants were conducted in accordance with the Declaration of Helsinki and Good Clinical Practice guidelines. Patients’ data were collected retrospectively from institutional records after ethics committee approval was obtained.

The datasets analyzed during the current study are not publicly available due to institutional data privacy policies but are available from the corresponding author upon reasonable request.

Generative AI tools, including ChatGPT (OpenAI, GPT-4, 2024 release) and Grammarly (version 1.0.40, 2024 desktop application), were used solely for language refinement and grammar correction. No AI assistance was involved in study design, data collection, statistical analysis, or interpretation.

**Figure 1:**
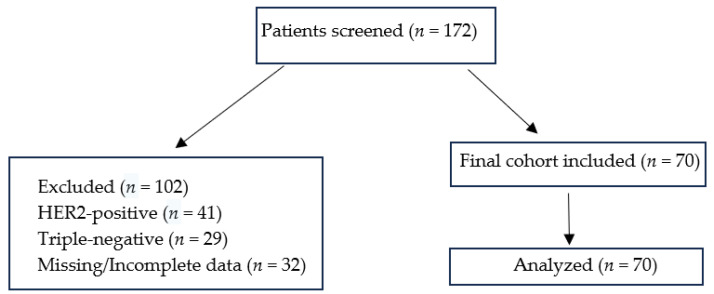
Flow diagram of patient selection and inclusion in the study.

A flow diagram illustrating patient selection, exclusions, and the final study cohort (*n* = 70) has been provided as Figure 1. A total of 172 patients with metastatic breast cancer were screened. Of these, 41 patients with HER2-positive disease, 29 patients with triple-negative disease, and 32 patients with missing or incomplete data were excluded. The final cohort comprised 70 patients with HR+/HER2− or HER2-low de novo metastatic breast cancer, all of whom were included in the analysis.

## 3. Results

### 3.1. Patient Characteristics

A total of 70 premenopausal and postmenopausal women with HR+, human epidermal growth factor HER2− or HER2-low de novo metastatic breast cancer were included in the analysis. The median age at diagnosis was 51 years (range: 33–77 years).

At the time of CDK4/6 inhibitor initiation, 53 patients (75.7%) were postmenopausal and 17 patients (24.3%) were premenopausal. The majority had an ECOG performance status of 0 (*n* = 56, 80.0%), while 14 patients (20.0%) had a score of 1.

HER2 status was classified according to IHC assessment. Patients with HER2 IHC 0 were considered HER2-negative (*n* = 33, 47.1%), while those with HER2 IHC 1+ or IHC 2+ and negative in situ hybridization (ISH) were categorized as HER2-low (*n* = 37, 52.9%).

In terms of CDK4/6 inhibitor use in the first-line setting, 37 patients (52.9%) received palbociclib and 33 (47.1%) received ribociclib. All patients had received a CDK4/6 inhibitor in combination with endocrine therapy (aromatase inhibitor or fulvestrant) as first-line treatment. In addition, each patient received at least one line of chemotherapy in the metastatic setting before everolimus initiation, most commonly taxane- or anthracycline-based regimens. Some patients received more than one line of chemotherapy, consistent with the definition of a heavily pretreated population.

Histologically, 37 patients (52.9%) had invasive ductal carcinoma, 26 (37.1%) had invasive carcinoma of no special type (NST), and 7 (10.0%) had invasive lobular carcinoma.

Based on the duration of prior CDK4/6 inhibitor therapy, patients were divided into two using the median treatment duration of 14.3 months. Thirty-five patients (50.0%) received CDK4/6 inhibitors for less than 14.3 months, while the remaining 35 patients (50.0%) received treatment for a longer duration.

The clinicopathological characteristics of the patients are summarized in Table 1.

### 3.2. Survival Outcomes

**Figure 2:**
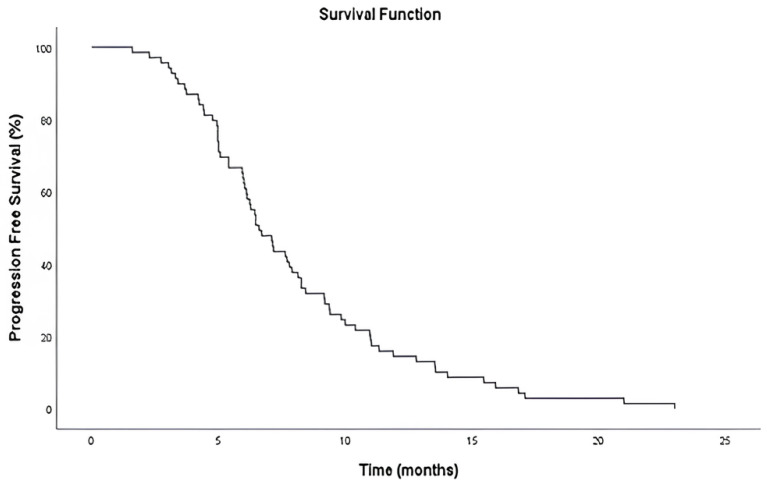
Kaplan–Meier curve illustrating PFS in patients treated with everolimus plus exemestane after prior CDK4/6 inhibitor and chemotherapy.

The median PFS was 6.6 months (95% CI: 5.59–7.61), as determined by Kaplan–Meier analysis (Figure 2). At the time of data cutoff, all 70 patients (100%) had experienced disease progression, and no patients were censored.

**Figure 3:**
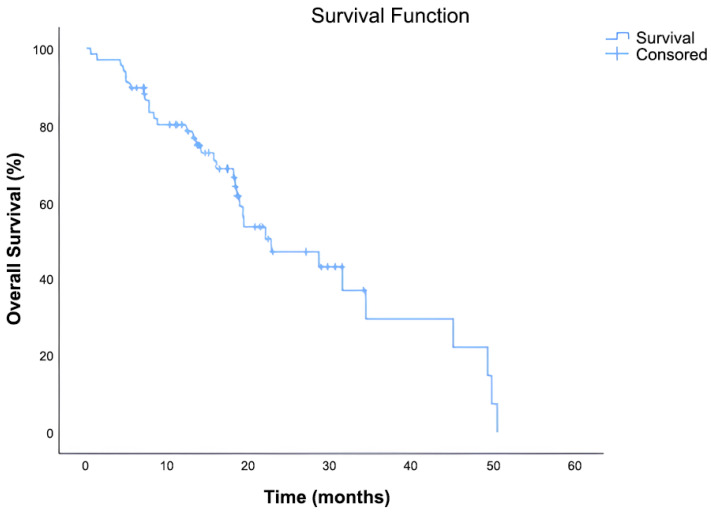
Kaplan–Meier curve illustrating OS in patients treated with everolimus plus exemestane after prior CDK4/6 inhibitor and chemotherapy.

The median OS was 22.6 months (95% CI: 11.92–33.35). Out of 70 patients, 36 (51.4%) had died, while 34 (48.6%) were censored at the time of data cutoff. The Kaplan–Meier survival curve demonstrated a continuous decline in survival probability over time, with approximately 30% of patients remaining alive at 36 months (Figure 3).

Univariate Cox regression analysis was performed to assess the impact of clinical and pathological variables on PFS. None of the tested variables including HER2 status (HER2-low vs. HER2-negative), menopausal status, ECOG performance status, histological subtype, type of CDK4/6 inhibitor received, and duration of CDK4/6 inhibitor use were found to be significantly associated with PFS in univariate analysis (all *p* > 0.05).

These variables were subsequently included in a multivariate Cox regression model. Similarly, no independent association with PFS was observed for any of the included covariates in the multivariate analysis.

Although both premenopausal (n = 17) and postmenopausal (n = 53) patients were included, no significant differences in PFS or OS were observed between these groups in subsequent analyses (all *p* > 0.05).

A detailed summary of the univariate and multivariate regression results is provided in Table 2.

Univariate Cox regression analysis revealed no statistically significant association between OS and the assessed clinical variables, including HER2 status, menopausal status, ECOG performance score, type of CDK4/6 inhibitor used, or duration of CDK4/6 inhibitor therapy. Consistently, none of these variables remained significant in the multivariate Cox regression model.

A detailed summary of these analyses is presented in Table 3.

### 3.3. Tumor Response and Disease Control

**Figure 4:**
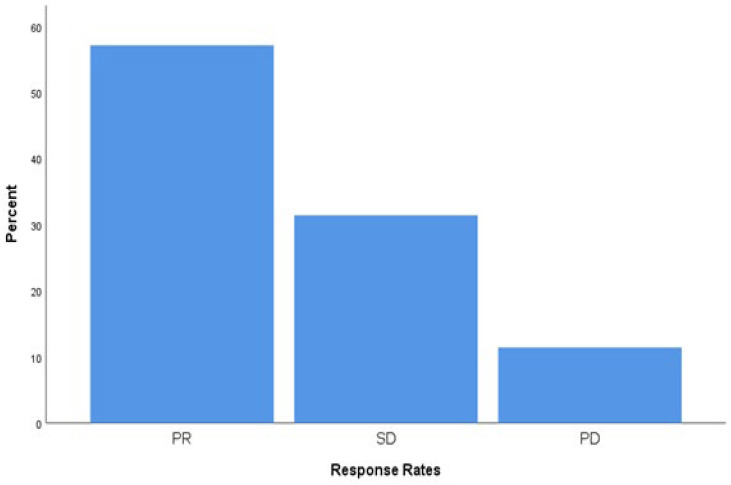
Bar chart showing the distribution of best radiologic responses among patients treated with everolimus plus exemestane. Abbreviations: PR, Partial response; SD, Stable Disease; PD, Progressive Disease.

Tumor response was evaluated radiologically according to RECIST criteria. Among the 70 patients included in the study, partial response (PR) was observed in 40 patients (57.1%), stable disease (SD) in 22 patients (31.4%), and progressive disease (PD) in 8 patients (11.4%). The disease control rate (DCR), defined as the combined proportion of PR and SD, was 88.6%. Response categories and their distribution are presented in Figure 4.

### 3.4. Adverse Events

Adverse events were recorded retrospectively based on clinical documentation. Among the 70 patients included in the analysis, no adverse events were reported in 38 patients (54.3%). The most frequently reported treatment-related side effect was fatigue (*n* = 14, 20.0%), followed by skin-related toxicity (*n* = 6, 8.6%) and stomatitis (*n* = 4, 5.7%). Other less common adverse events included diarrhea (*n* = 3, 4.3%), allergic reactions (*n* = 2, 2.9%), and hematologic toxicity (*n* = 1, 1.4%). Edema, pneumonitis, and hematologic toxicity were each reported in one patient (1.4%). No treatment-related deaths were documented, and no patients discontinued therapy due to toxicity. In line with the retrospective design, adverse events were recorded from available documentation, and no dose modifications or treatment interruptions were reported. A detailed summary of the adverse events is presented in Table 4.

## 4. Discussion

In this retrospective, single-center study, we evaluated the real world efficacy and safety of everolimus in combination with exemestane in a cohort of postmenopausal and premenopausal women with HR+/HER2− metastatic breast cancer who had previously received CDK4/6 inhibitor therapy and at least one line of chemotherapy. One of the main mechanisms responsible for endocrine resistance in HR+ breast cancer is the activation of the PI3K/AKT/mTOR pathway. This signaling pathway plays a key role in regulating cell growth, survival, and metabolism. When activated, it can stimulate estrogen receptor (ER) activity even in the absence of estrogen, contributing to resistance against hormonal treatments. Everolimus is a selective mTOR inhibitor that blocks this pathway by binding to mTOR complex 1 (mTORC1). This inhibition prevents the activation of downstream proteins such as S6 kinase and 4EBP1, which are involved in cancer cell growth and proliferation. Preclinical studies have shown that everolimus can restore hormone sensitivity and enhance the effect of endocrine therapy in resistant tumors [11].

The efficacy outcomes observed in our study are in line with those reported in the pivotal BOLERO-2 trial, which demonstrated a significant improvement in PFS with the addition of everolimus to exemestane in postmenopausal women with HR+/HER2− advanced breast cancer previously treated with nonsteroidal aromatase inhibitors. In BOLERO-2, everolimus plus exemestane achieved a median PFS of 6.9 months (local assessment) compared to 2.8 months in the placebo group (HR: 0.43; *p* < 0.001) [6]. In our real-world cohort, comprising patients who were more heavily pretreated, including prior CDK4/6 inhibitors and chemotherapy the median PFS was 6.6 months, which is comparable despite the more advanced treatment line. This suggests that everolimus retains clinical efficacy even in patients who have progressed beyond current standard first line treatments. While direct comparisons are limited by differences in study design and patient populations, our results reinforce the clinical benefit of mTOR inhibition in HR+/HER2− metastatic breast cancer and support its continued use in later lines of therapy under real-world conditions.

One of the most noteworthy aspects of the BOLERO-6 trial was the inclusion of capecitabine as a comparator, offering valuable insights into the choice between targeted therapy and chemotherapy in the endocrine-resistant setting. In BOLERO-6, capecitabine yielded a median PFS of 9.6 months, which was numerically longer than that of the everolimus plus exemestane arm (8.4 months); however, this difference was not statistically tested and was influenced by baseline imbalances and potential informative censoring [12]. In our real-world cohort, patients had received both prior CDK4/6 inhibitors and at least one line of chemotherapy before everolimus-based therapy, representing a more heavily pretreated group. Despite this, the median PFS observed with everolimus plus exemestane in our study was 6.6 months, which remains clinically meaningful. These findings suggest that, even in later treatment lines, everolimus plus exemestane retains its activity and may be considered a viable option in patients who are not ideal candidates for further cytotoxic chemotherapy or wish to avoid its toxicity profile.

The EMERALD trial demonstrated the efficacy of elacestrant, a novel oral SERD, in patients with ER-positive, HER2-negative advanced breast cancer who had progressed after prior endocrine therapy and CDK4/6 inhibitors. In this phase 3 trial, which included both ESR1-mutant and wild-type populations, the median PFS with elacestrant was 2.8 months in the overall population and 3.8 months in patients with ESR1 mutations [13]. In contrast, our real-world study, which included a more heavily pretreated population, having received both CDK4/6 inhibitors and at least one line of chemotherapy reported a median PFS of 6.6 months with everolimus plus exemestane. While cross trial comparisons should be interpreted cautiously, this numerical difference highlights the sustained activity of mTOR inhibition even in later lines of therapy. Our findings support the use of everolimus-based combinations as a valuable treatment option beyond CDK4/6 inhibitors, particularly in patients who have already received chemotherapy or do not harbor ESR1 mutations, where the efficacy of SERDs may be limited.

The SOLAR-1 trial led to the approval of alpelisib plus fulvestrant for patients with PIK3CA-mutated, HR+/HER2− advanced breast cancer. However, only about 6% of the patients in that trial had previously received CDK4/6 inhibitors, which limits how well its results apply to current treatment practice [14]. To address this, the BYLieve trial was designed as a phase 2, single-arm study including patients who had already progressed on CDK4/6 inhibitors. In cohort A, the median PFS with alpelisib plus fulvestrant was 7.3 months [15]. In our real-world study, patients were more heavily pre-treated, including chemotherapy in addition to CDK4/6 inhibitors and achieved a median PFS of 6.6 months with everolimus plus exemestane. Although our population was not selected by PIK3CA mutation status, and these trials are not directly comparable, the similar PFS outcomes are still informative. These results, together with the findings from the BOLERO-2 and BOLERO-6 trials, suggest that mTOR inhibition remains a valid treatment approach in the endocrine-resistant setting, especially in cases where genomic testing is unavailable or PI3K inhibitors are not accessible. While these studies differ in design, the lack of a major PFS difference supports the continued clinical use of everolimus-based combinations.

Recent studies have also explored the combination of everolimus and exemestane with CDK4/6 inhibitors. The TRINITI-1 trial evaluated the triplet regimen of ribociclib, everolimus, and exemestane in HR+/HER2− advanced breast cancer patients who had progressed on prior CDK4/6 inhibitor therapy. The study reported a clinical benefit rate of 41.1% at 24 weeks and a median PFS of 5.7 months, despite the continuation of CDK4/6 inhibition beyond progression [16]. Another recent multiomic phase Ib/II study combined palbociclib, everolimus, and exemestane in a heavily pretreated cohort, including a high proportion of ESR1-mutated tumors, and reported a clinical benefit rate of only 18.8% [17]. In contrast, our real-world study, which did not include CDK4/6 inhibitor continuation and enrolled a molecularly unselected population, demonstrated a median PFS of 6.6 months and a disease control rate of 88.6%. While these findings are not from head-to-head comparisons, they suggest that everolimus plus exemestane may retain significant efficacy even without concurrent CDK4/6 inhibition, particularly in later lines of treatment where maintaining tolerability is critical.

The safety profile observed in our study was largely consistent with the known toxicity spectrum of everolimus plus exemestane reported in the BOLERO-2 trial [6]. In that pivotal study, the most common treatment-related adverse events included stomatitis (59%), fatigue (37%), rash (39%), diarrhea (34%), and hyperglycemia (14%), with grade 3–4 stomatitis occurring in approximately 8% of patients. In comparison, our real-world cohort demonstrated a lower incidence of reported adverse events, with fatigue (20.0%), skin-related toxicity (8.6%), and stomatitis (5.7%) being the most frequently documented. Importantly, no grade 3–4 toxicities or treatment-related deaths were recorded in our study, and no patients discontinued therapy due to toxicity. However, this apparent discrepancy may be partly explained by the retrospective nature of data collection, which can lead to underreporting of non-severe or subclinical toxicities, especially in settings lacking systematic CTCAE-based adverse event grading. Despite this limitation, the absence of unexpected safety signals and the relatively manageable toxicity profile observed in our cohort reinforce the feasibility of using everolimus plus exemestane in daily clinical practice, particularly for patients who have already received CDK4/6 inhibitors and chemotherapy.

Interestingly, in both univariate and multivariate analyses, no statistically significant associations were observed between clinical or pathological variables including HER2 status, menopausal status, ECOG performance score, CDK4/6 inhibitor type or duration and progression free or overall survival. The number of events in the OS analysis (36 deaths) was relatively limited, which may affect the precision of the multivariate estimates. While such findings might suggest a lack of predictive markers for treatment response, they may also reflect the broadly consistent efficacy of everolimus plus exemestane across diverse patient subgroups. This observation is particularly relevant given the single-arm design of our study and the real-world setting, where heterogeneity in baseline characteristics is common. The absence of significant subgroup differences reinforces the notion that this combination therapy may offer clinical benefit to a wide spectrum of HR+/HER2− metastatic breast cancer patients who have progressed following CDK4/6 inhibition and chemotherapy.

However, some limitations of our study should be considered. Since this was a retrospective, single-center study, there may have been selection bias, and our results may not fully reflect the broader patient population. Also, we did not have access to detailed molecular data such as PIK3CA or ESR1 mutation status, which could have helped identify which patients benefit most from everolimus-based treatment. Another important point is the lack of a control group, which makes it difficult to compare our results directly with other treatment options. Because the study was performed in a real-world setting, adverse events may not have been recorded completely, especially if they were mild. Despite these limitations, our findings provide useful information about the effectiveness and tolerability of everolimus plus exemestane in daily clinical practice, especially in patients who have already received CDK4/6 inhibitors and chemotherapy. Further prospective studies with larger patient numbers and biomarker analysis are needed to guide more personalized treatment decisions.

## 5. Conclusions

In this real-world, retrospective study, the combination of everolimus and exemestane demonstrated meaningful clinical activity in patients with HR+/HER2− metastatic breast cancer who had progressed after both CDK4/6 inhibitors and chemotherapy. The observed progression-free survival and disease control rates were comparable to those reported in clinical trials, despite a more heavily pretreated population. No significant associations were identified between survival outcomes and baseline clinical features, suggesting a potential benefit across a broad patient spectrum. These findings support the continued use of everolimus plus exemestane as a valuable treatment option in later lines of therapy, particularly in settings where access to newer targeted agents is limited. Prospective studies incorporating molecular profiling are warranted to further optimize patient selection and outcomes.

## Figures and Tables

**Table 1:** Baseline clinicopathological characteristics of the patients.

Variable	*n* (%)
**Median age at diagnosis, years (range)**	51 (33–77)
**Menopausal status**	
Postmenopausal	53 (75.7%)
Premenopausal	17 (24.3%)
**ECOG performance status**	
0	56 (80.0%)
1	14 (20.0%)
**HER2 status**	
HER2-negative (IHC 0)	33 (47.1%)
HER2-low (IHC 1+ or 2+/ISH−)	37 (52.9%)
**Histological type**	
Invasive ductal carcinoma	37 (52.9%)
Invasive carcinoma NST	26 (37.1%)
Invasive lobular carcinoma	7 (10.0%)
**Type of CDK4/6 inhibitor used**	
Palbociclib	37 (52.9%)
Ribociclib	33 (47.1%)
**Duration of CDK4/6 inhibitor use**	
<14.3 months	35 (50.0%)
≥14.3 months	35 (50.0%)

Abbreviations: HER2, human epidermal growth factor receptor 2; IHC, immunohistochemistry; CDK4/6, cyclin-dependent kinase 4 and 6; ISH, in situ hybridization.

**Table 2:** Univariate and Multivariate Cox Regression Analysis for Progression-Free Survival.

Variable	HR (Univariate)	95% CI (Univariate)	*p*-Value (Univariate)	HR (Multivariate)	95% CI (Multivariate)	*p*-Value (Multivariate)
HER2 status (Low vs. negative)	0.70	0.43–1.13	0.148	0.68	0.40–1.16	0.160
Menopausal status	0.97	0.55–1.70	0.906	1.11	0.61–2.02	0.744
ECOG performance status	1.04	0.58–1.89	0.884	0.79	0.41–1.53	0.488
Histological subtype	0.86	0.67–1.11	0.241	0.91	0.66–1.26	0.568
Type of CDK4/6 inhibitor	1.36	0.83–2.21	0.218	1.44	0.87–2.39	0.155
Duration of CDK4/6 inhibitor	1.23	0.76–1.99	0.392	1.20	0.70–2.06	0.500

Abbreviations: HR, hazard ratio; CI, confidence interval; CDK4/6, cyclin-dependent kinase 4 and 6; ECOG, Eastern Cooperative Oncology Group.

**Table 3:** Univariate and Multivariate Cox Regression Analysis for Overall Survival.

Variable	HR (Univariate)	95% CI (Univariate)	*p*-Value (Univariate)	HR (Multivariate)	95% CI (Multivariate)	*p*-Value (Multivariate)
HER2 status (Low vs. Negative)	1.55	0.75–3.21	0.238	1.46	0.68–3.12	0.331
Menopausal status	1.25	0.56–2.77	0.588	1.30	0.57–2.97	0.530
ECOG performance status	0.86	0.32–2.26	0.754	0.84	0.29–2.39	0.740
Duration of CDK4/6 inhibitor use	0.96	0.48–1.94	0.910	1.18	0.55–2.54	0.675
Type of CDK4/6 inhibitor	1.60	0.75–3.41	0.223	1.61	0.74–3.53	0.233

Abbreviations: HR, hazard ratio; CI, confidence interval; CDK4/6, cyclin-dependent kinase 4 and 6; ECOG, Eastern Cooperative Oncology Group.

**Table 4:** Summary of Adverse Events.

Adverse Event	Number of Patients (*n*, %)
None	38 (54.3%)
Fatigue	14 (20%)
Edema	1 (1.4%)
Stomatitis	4 (5.7%)
Skin toxicity	6 (8.6%)
Pneumonitis	1 (1.4%)
Allergy	2 (2.9%)
Hematologic toxicity	1 (1.4%)
Diarrhea	3 (4.3%)

## Data Availability

While the datasets analyzed in this study are not publicly accessible, they can be obtained from the corresponding author upon reasonable request.

## Abbreviations

The following abbreviations are used in this manuscript:

HR+	Hormone receptor positive
HER2	Human epidermal growth factor receptor 2 negative
MBC	Metastatic breast cancer
SERMs	Selective estrogen receptor modulators
SERDs	Selective estrogen receptor degraders
AIs	Aromatase inhibitors
PI3K	Phosphatidylinositol 3-kinase
AKT	Protein kinase B
mTOR	Mammalian target of rapamycin
HIF-1α	Hypoxia-inducible factor 1-alpha
CDK4/6	Cyclin-dependent kinase 4 and 6
IHC	Immunohistochemistry
ISH	In situ hybridization
ECOG	Eastern Cooperative Oncology Group
PFS	Progression-free survival
OS	Overall survival
RECIST	Response Evaluation Criteria in Solid Tumors
HR	Hazard ratio
CI	Confidence interval
PR	Partial response
SD	Stable disease
PD	Progressive disease
DCR	Disease control rate
ER	Estrogen receptor
ESR1	Estrogen receptor 1
NST	No special type (as in invasive carcinoma NST)
